# A Straightforward Method for Adipocyte Size and Count Analysis Using Open-source Software QuPath

**DOI:** 10.1080/21623945.2022.2027610

**Published:** 2022-01-30

**Authors:** Ville A Palomäki, Vesa Koivukangas, Sanna Meriläinen, Petri Lehenkari, Tuomo J Karttunen

**Affiliations:** a Department of Surgery, Oulu University Hospital and University of Oulu, Oulu, Finland; b Cancer and Translational Medicine Research Unit, Medical Research Center Oulu, Oulu University Hospital and University of Oulu, Oulu, Finland

**Keywords:** Adipocyte size, fat cell size, Qupath, Adipocyte Tools, ImageJ, obesity

## Abstract

Changes in adipose tissue morphology, depicted by cell morphology alterations such as enlargement of fat cells, always accompany over-weight and obesity. The variables related to cell size have been shown to associate with low-grade inflammation of adipose tissue and common obesity-related comorbidities including metabolic syndrome and type 2 diabetes. Quantifying fat cell morphology from images of histological specimens can be tedious. Here, we present a straightforward method for the task using the free open-source software QuPath with its inbuilt tools only. Measurements of human adipose tissue samples with the described protocol showed an excellent correlation with those obtained with ImageJ software with Adipocyte Tools plugin combined with manual correction of misdetections. Intraclass correlation between the two methods was at good to excellent level. The method described here can be applied to considerably large tissue areas, even whole-slide analysis.

## 1. Introduction

Obesity has become a pivotal health concern worldwide [1,2]. A link between excess weight and comorbidities, such as metabolic syndrome and type 2 diabetes is well established and seems largely to originate from dysfunctional adipose tissue harbouring low-grade inflammation [3–5]. The expansion of adipose depots is accompanied by adipocyte hypertrophy, i.e., enlargement of fat cell size (FCS), which reflects adipose tissue dysfunction and overall metabolic impairment [6]. For instance, in obesity, FCS correlates positively with the degree of macrophage infiltration, which is considered a marker of chronic inflammation [7–9]. FCS enlargement in subcutaneous adipose tissue (SAT) reflects insulin resistance and fat accumulation in the liver as well [10–13]. Assessing the hyperplasia-hypertrophy-ratio and population of smaller adipocytes may be of importance as well [14]. In this view, quantitative description of adipose tissue morphology, such as FCS and cell density can be used indirectly to address tissue dysfunction and inflammation. For instance, FCS and cell density could be utilized to measure the effects of medical or surgical interventions of obesity. Thus, there is a call for an accurate, convenient, and reproducible method to assess adipocyte size and count.

The currently available techniques to measure FCS are commonly based on flow cytometry after collagenase treatment or direct histological analysis [6,15]. Histological analysis may, to some extent, evade biases due to cell rupture in cytometric techniques and allows simultaneous assessment of tissue morphology such as crown-like structures associated with dead or dying adipocytes [7]. A conventional way to assess histological samples has been micrograph quantification, i.e., measuring adipocytes from a photograph taken with a camera attached to a microscope [6]. However, affordable and accurate slide scanners have since made it easy to digitize whole-slide images for further inspection [16].

Assessing large amounts of adipocytes in histological samples can be cumbersome. Commercial software for this purpose can be expensive and awkward to use [16]. However, open-source alternatives such as ImageJ with numerous published scripts and plugins are available [17,18]. Adipocyte Tools is a commonly used plugin and reliable for micrograph quantification [16,19]. However, high-resolution whole-slide scanned images may reach up to 2 GB and cannot be assessed directly as such due to restrictions in file size. On the contrary, the novel and increasingly popular open-source software QuPath can handle larger images [20]. In-house plugins and scripts for adipocyte measurement have been developed for QuPath as well [16,21].

We present here a simple, straightforward and fast method for FCS measurement using QuPath with only its inbuilt tools, with no need for external plugins or scripts. Measurements are compared to those obtained with ImageJ + Adipocyte Tools plugin with manual correction of any misdetection in conditions mimicking conventional photomicrograph quantification. The method described can be extended to whole-slide measurement of adipose tissue sections containing up to at least 12,000 cells as demonstrated here.

## 2. Results

### 2.1. QuPath and ImageJ with adipocyte tools plugin in the detection of fat cells

**Figure 1.**
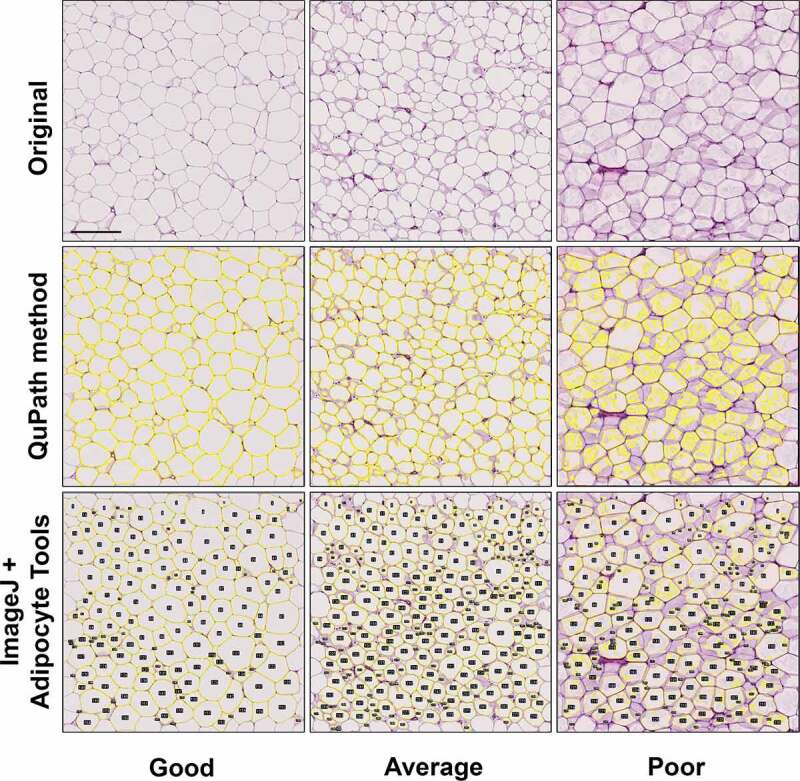
Comparison of the ability of QuPath and ImageJ plugin Adipocyte Tools to detect the adipocytes in images of H&E stained 5 µm adipose tissue sections. Section quality and concomitant success of correctly rendering the cell area was graded on a visual scale of good-average-poor. See text for details. Scale bar 200 µm.

**Figure 2.**
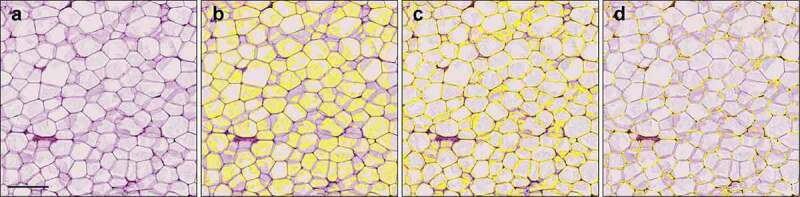
Comparison of alternate pixel classifiers in illustrative cases of poor quality (H&E) histological samples. For size measurement, rendering the seemingly empty adipocyte area correctly is crucial and spillover of staining results in difficulties of the QuPath’s pixel classifier to appropriately detect the cell area (b-d). Training the classifier to recognize weakly stained spillover may help, but sometimes it results in inclusion of weakly stained sections of cell membranes, resulting in detection of falsely conjoined cells (c), or even reversal of detections (d). Scale bar 200 µm.

For the QuPath protocol, a pixel classifier to distinguish non-stained areas representing mainly dissolved fat within the fat cells and stained areas representing fat cell walls and cytoplasm and other tissue components was trained using a good quality section (see Materials and methods for details). Visual evaluation of the samples with the classifier showed that section and staining quality determined the success of rendering the adipocytes (Figure 1). Section quality and concomitant success of rendering adipocytes with QuPath was visually assessed with an arbitrary scale as good, average or poor as illustrated in Figure 1. In most samples (52/60), section quality and the concomitant rendering were good to average, with all (good) or great majority (average) of adipocyte cell membranes being thin and crisply stained, and the extent of the fat area seemed correctly identified by the pixel classifier. A minority (8/60) of the samples were classified as poor. These specimens showed faintly stained areas adjacent to fat cell membranes partially filling the cells (Figure 1). The exact mechanisms for this kind of staining could not be addressed, but their background is likely artefactual and related to tissue processing including fixation, section thickness and possibly staining conditions. In these samples, the QuPath method typically could not detect the cell boundaries correctly due to excessive spilling of staining (Figure 1). Training of the pixel classifier by annotating faintly stained intracellular areas improved correct cell identification in some samples with quality classified as poor, but simultaneously, led to improper detection of adjacent cells as conjoined cells, or even reversal of detection (Figure 2). Finally, samples representing average to good level when assessed with the selected classifier were further analysed.

**Figure 3.**
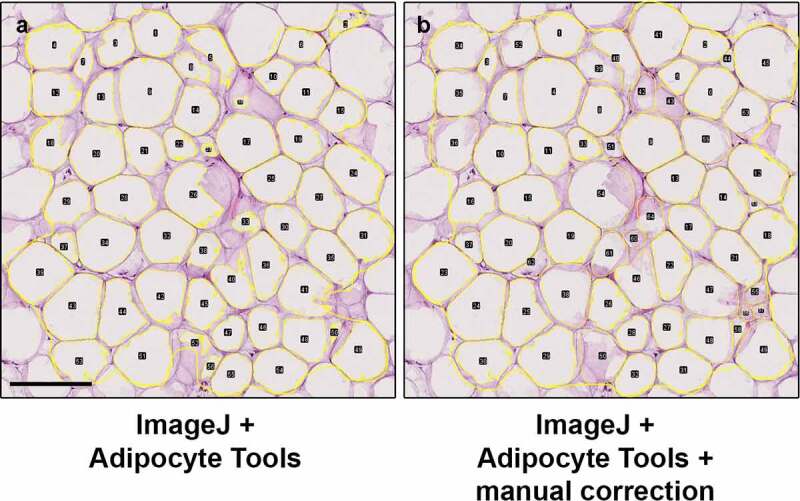
ImageJ plugin Adipocyte Tools rendered fat cell detections (a) can be manually corrected (b) if necessary, effectively resulting in delineation of cells equivalent to full manual detection. With the QuPath protocol, there are similar options. Scale bar 200 µm.

The ImageJ plugin Adipocyte Tools rely similarly on detecting areas of dissolved fat in sections. As demonstrated in third row of Figure 1, poor quality specimens frequently result in misdetection of cell boundaries with Adipocyte Tools as well. However, the ImageJ allows the deletion of detections and manual redrawing of the boundaries when necessary, effectively resulting in detections equivalent to full manual delineation of adipocytes (Figure 3). The results of measurements obtained using ImageJ + Adipocyte Tools with manual correction of any misdetections are henceforth used as basis of comparison.

### 2.2. Comparison of performance of adipocyte tools with manual correction and QuPath in measuring adipocytes

**Figure 4.**
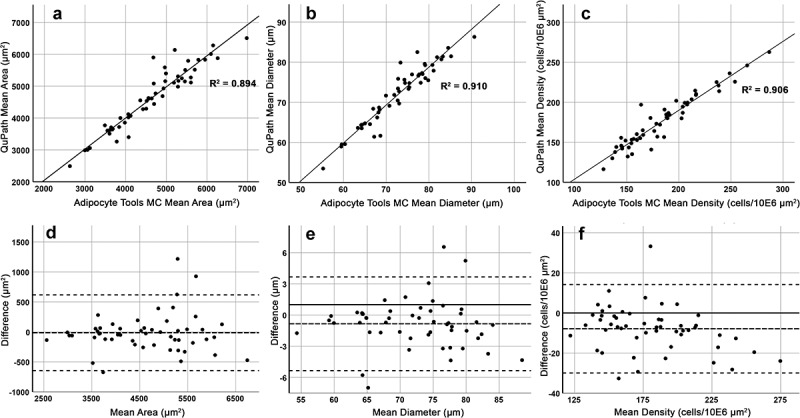
Adipocyte Tools with manual correction (MC) and QuPath based methods show good intermethod consistency. The QuPath based method measured on average 13.7 µm^2^ smaller area, 0.8 µm smaller diameter, and 7.9 higher density (cells/10E6 µm^2^). A-C: Scatter-plots with linear regression for mean area, mean diameter and mean density. D-F: Respective Bland-Altman plots with middle dashed-line set at mean difference level.

**Figure 5.**
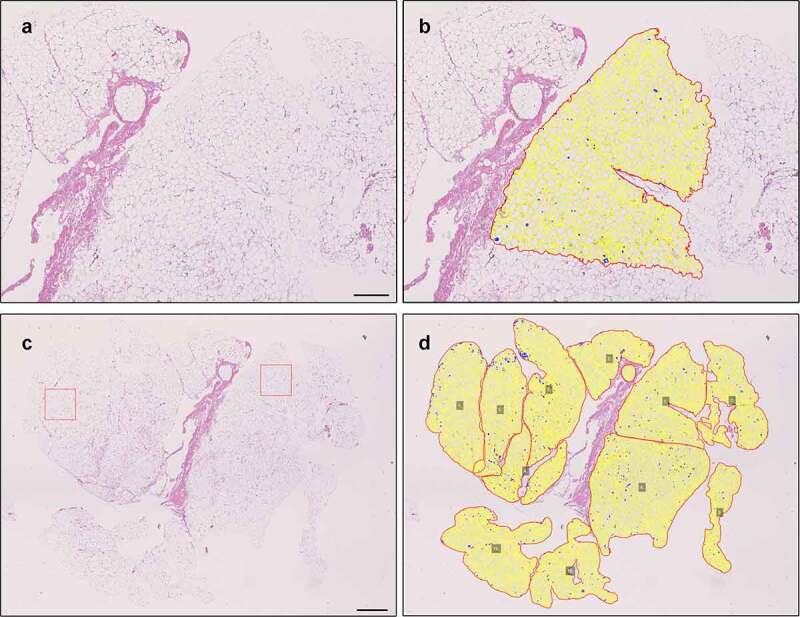
The QuPath based method is applicable for analysis of large adipocyte areas as well. A-B: Illustration of QuPath-based method finding the adipocytes in a larger area (7.1x10E6 µm^2^). Scale bar 500 µm. C-D: Analysing the whole slide requires more effort but can be achieved in pieces on an average powered workstation (total area 62x10E6 µm^2^). Red boxes (10E6 µm^2^) demonstrate areas used for basic analyses with ImageJ + Adipocyte Tools plugin. Scale bar 1,000 µm.

Adipocyte count, mean area, mean diameter and cell density were measured in 2x10E6 µm^2^ areas extracted from representative adipocyte areas (exemplified with rectangles in Figure 5c) of 52 samples estimated at average or good level according to the visual scale. In these samples, the QuPath-based method found on average 304 ± 60 fat cells compared with 327 ± 67 detected with Adipocyte Tools and manual correction. The measurements obtained with QuPath deviated only slightly from those with Adipocyte Tools with manual correction. On average, the area and diameter measured by QuPath was 13.7 µm^2^ (0.3%) and 0.8 µm (1.1%) smaller, respectively. In scatter plots with linear regression and Bland-Altman plot modified to set on the difference, the consistency of the measurements between these two methods was good (Figure 4). The intraclass coefficients (ICC) based on two-way mixed-effect model and aimed at consistency were calculated for inter-method comparison. The ICCs and respective 95% confidence intervals were in a good range for the number of adipocytes found and in excellent range for area, diameter and density measurements (Table 1).

**Table 1.** Intraclass Correlation Coefficients and respective 95% Confidence Intervals for average and good samples. Two-way mixed effect model aimed for consistency. Single measures

	Variable	n	ICC	95% CI
Adipocyte Tools + manual correction vs QuPath	Count **	52	0.900	0.831 to 0.941
	Area ***	52	0.945	0.906 to 0.968
	Diameter ***	52	0.954	0.921 to 0.973
	Density ***	52	0.945	0.906 to 0.968

F test for all ICC values < .000.

ICC = Intraclass Correlation Coefficient.

CI = Confidence Interval for ICC.

Reliability according to lower CI boundary:

- Good **

- Excellent ***

### 2.3. QuPath Based Method Successfully Analyses Large Adipocyte Areas and Whole Slides

As noted, QuPath can handle larger images more conveniently than ImageJ, as the latter requires importing series of separate images. The method based on QuPath described here thus allows fast measurement of larger areas. To demonstrate this, we measured 21 larger adipocyte areas (size range 4.9–17.8 x10E6 µm^2^, mean 9.4x10E6 µm^2^ ± 3.5x10E6 µm^2^) as illustrated in Figure 5a and 5b. The results are summarized in Table 2B.

**Table 2.** (A) Comparison of ImageJ with Adipocyte Tools plugin and QuPath for adipocyte count, area, diameter and density according to quality of staining in 40 subjects with BMI > 35 kg/m^2^. Adipocyte Tools was used both with and without manual correction. (B) Example of substantially larger areas measured with QuPath method in subjects with BMI > 35 kg/m^2^

A (2x10E6 µm^2^)	n	Count	Mean area (µm^2^)	Mean diameter (µm)	Mean density (cells/10E6 µm^2^)
ImageJ + Adip. Tools with manual correction (all)	40	315.5 ± 71.7	4827 ± 1290	74.0 ± 9.9	179.6 ± 39.8
- Good	14	324.9 ± 66.4	4906 ± 959	74.8 ± 7.6	182.8 ± 35.0
- Good + Average	34	321.7 ± 68.2	4754 ± 951	73.4 ± 7.6	182.0 ± 34.9
- Poor	14	324.9 ± 66.4	4906 ± 959	74.8 ± 7.6	182.8 ± 35.0
ImageJ + Adip. Tools no manual correction (all)	40	283 ± 63.1	4585 ± 1100	71.5 ± 8.6	176.9 ± 46.6
- Good	14	277.8 ± 54.0	4882 ± 883	74.0 ± 6.9	171.0 ± 29.3
- Good + Average	34	286.1 ± 58.6	4639 ± 950	72.0 ± 7.5	179.4 ± 45.5
- Poor	6	264.8 ± 89.0	4279 ± 1828	68.3 ± 14.0	162.7 ± 54.3
QuPath (all)	40	294.5 ± 63.2	4657 ± 1189	71.8 ± 9.3	172.5 ± 36.6
- Good	14	293.0 ± 58.0	5034 ± 917	74.8 ± 6.7	170.7 ± 31.3
- Average + Good	34	299.2 ± 59.4	4746 ± 1009	72.6 ± 7.7	174.3 ± 33.2
- Poor	6	267.8 ± 82.6	4151 ± 1986	66.7 ± 15.6	162.0 ± 55.1
B (5–18x10E6 µm^2^)					
QuPath – large area good + average	21	1613.9	4695 ± 1020	71.8 ± 7.7	175.9 ± 42.6

Values are mean ± SD.

Good, Average and Poor indicate class of section and staining quality.

Analysis of a whole-slide image is demonstrated in Figure 5c and 5d. A total of 11,964 adipocytes were found. Mean area, diameter and density of the fat cells are summarized in Table 3 along with those measured in smaller sample areas of that slide used in method validation and a larger sample area (Figure 5b). We noticed that at least on an average-powered workstation, attempts to analyse areas over 20x10E6 µm^2^ in size might result in software freeze. Thus, the whole-slide analysis had to be done combining several smaller measured sample areas as demonstrated (Figure 5d).

**Table 3.** Comparison of ImageJ with Adipocyte Tools plugin (with manual correction) and QuPath protocol for adipocyte count, mean area, mean diameter and density in one sample (Figure 5). Two small areas (A) and large area or whole slide (B) were measured

A	Count	Mean area (µm^2^)	Mean diameter (µm)	Density (cells/10E6 µm^2^)
*ImageJ + Adipocyte Tools + manual correction	303	4907 ± 2914	75.2 ± 24.28	172.7
*QuPath	310	4687 ± 2858	73.3 ± 24.31	180.3
B				
QuPath (large area)	1389	4134 ± 3303	68.1 ± 25.1	198.1
QuPath (whole-slide)	11,964	4140 ± 4049	67.4 ± 27.1	193.8

Values are mean ± SD for Area and Diameter.

* Same two 1000 µm x 1000 µm areas measured with both methods.

### 2.4. Comparison of the Methods in the Group of Obese Subjects

Finally, ImageJ with Adipocyte Tools plugin and our QuPath protocol were utilized to measure adipocyte count, area, diameter and density in a population of 40 subjects with BMI > 35 kg/m^2^. For measurement, 2x10E6 µm^2^ areas from the H&E-stained sections were used. The QuPath protocol was also used to measure larger (on average 9.4x10E6 µm^2^) samples fulfiling the same BMI criteria. Cell measurements indicated that overall, the QuPath protocol and ImageJ + Adipocyte Tools provide comparable results, both within the range previously reported for such high BMI subjects [6] (Table 2). Along with decreasing section and staining quality, both methods provide similarly lower adipocyte measures, likely related with comparable inclusion of adipocytes with artefactually small measurement results, as described in section 2.1 and in Figures 1–3.

## 3. Materials and methods

### 3.1. Image analysis

Archived high-resolution whole-slide images of Haematoxylin and Eosin (H&E) stained adipose human tissue sections from other current projects of our group were used. The original tissue specimens were acquired from periumbilical subcutaneous adipose tissue of morbidly obese patients (BMI 39.01 kg/m^2^ ± 5.67 kg/m^2^, lowest 28.55 kg/m^2^ and highest 53.01 kg/m^2^, BMI information missing for 7 patients) participating in a study comparing the effect of bariatric surgery vs. conservative treatment on obesity-related comorbidities. The project for tissue specimen collection was approved by the local ethics committee (PPSHP:n alueellinen eettinen toimikunta) following the principles of the Declaration of Helsinki and informed written consent for participation was acquired. Specimens had been routinely fixed in formalin, processed to paraffin, sectioned at 5 µm and stained, and scanned with Leica Aperio AT2 (Leica Biosystems, Buffalo Grove, IL, USA) at 40x magnification. A total of 60 images was reviewed, and two 1000 µm x 1000 µm samples from homogeneous adipocyte areas of each image were sent to ImageJ [22] and further analysed with the Adipocyte Tools plugin using the option for large cells and the settings of minimum size 500 µm2 and number of erosions rounds 3 [23]. Faulty detections were manually corrected. Results were further processed in Excel-spreadsheet software (Microsoft Corporation, Redmond, Washington, USA). Cell diameter was calculated assuming circular shape of adipocytes and density was assessed using the area drawn around detected cells.

The same images were assessed with QuPath 0.2.3 [20]. First, a random trees-based pixel classifier was trained to detect two tissue components in the sections: 1) tissue area, mainly consisting of cell wall remnants, and 2) dissolved empty ‘fat’ areas within the adipocytes. Training involved annotation of both components until the classifier was able to detect them visually reliably (Figure 1) when tested and reviewed in several different images. The classifier could not, even with sets of images, be reliably trained to distinguish cell walls from other tissue in a way that would work for all images; instead, it had to be trained to distinguish empty areas from *any* tissue. Eventually, one clearly stained image was used for training and the result was evaluated with several images.

The final pixel classifier was based on Random detection pattern with high resolution and otherwise on QuPath’s default settings. The pixel classifier was then applied on an exactly annotated area consisting of whole adipocytes. We used QuPath’s brush and wand tools for annotation of area of interest. Detections made by the classifier were converted to annotation-type objects with the ‘Create objects’ command. After visually comparing different settings of the command for optimal detection of fat cells, we used the following settings: min object size 20 µm^2^, min hole size 30 µm^2^, split objects, delete existing objects, and set new objects to selected.

With QuPath, it is optionally possible to manually select and delete any detections such as those interpreted to represent misdetections. It is also possible to manually annotate such misdetected adipocytes.

QuPath automatically measures annotations including area, circumference and circularity, and tabulates all measurements. Measurements from each image were transferred to Excel where cell count, mean area and mean diameter were calculated based on desired area range (here we used area of 500–100,000 µm^2^). We used the spreadsheet-software’s commands COUNTIFS and AVERAGEIFS to pick the desired data.

### 3.2. Statistical analysis

Statistical analyses were performed and the graphs built using SPSS Statistics (version 25, IBM Corp., Armonk, NY, USA). Values are shown as mean plus or minus standard deviation (SD). Correlations were illustrated using scatter plots and Bland-Altman plots set to mean difference. Intraclass correlation coefficient estimates and their 95% confidence intervals were calculated based on two-way mixed-effects model aimed at consistency.

## 4. Discussion

We describe here a new simple and fast protocol for adipocyte size measurement by using whole-slide images scanned from H&E stained sections. For measurements, we use only the basic functions of an open access image analysis program, QuPath, with no need for additional programming, scripts or plugins. Measurements with our protocol provide data highly comparable with those obtained with Adipocyte Tools combined with manual correction of any misdetections. An additional benefit of our protocol includes the possibility to measure larger areas of samples as compared to snaps of limited size usable in Adipocyte Tools and other ImageJ plugins.

The concept of the present method is based on QuPath’s pixel classifier, a machine learning algorithm for the detection of different areas within images. The classifier is first trained by annotating tissue area types to be detected; here, firstly, the empty spaces within the fat cells and secondly, cell walls and other components stained with H&E. The machine learning algorithm then builds up a set of optimal criteria for the automatic recognition of these tissue components. A handy feature in the program is the possibility to further improve the performance of the classifier by annotating any misclassified regions. Finally, the optimally functioning classifier can be saved and used for the analysis of case series. After running the optimized pixel classifier, we used the ‘Create objects’ option in QuPath, by which it is possible to transform each empty space in fat cells to an object with numerical data about area and circumference. This algorithm has several adjustable settings, such as object size range, which can be adjusted for optimal inclusion of fat cells. Finally, QuPath’s algorithms allowed reliable detection of fat cells and measurement of the count and the area of adipocytes in the desired region. It should be noted that although QuPath is a flexible and competent program for quantitative cell analyses, its basic cell detection algorithms are not suitable for adipocytes. This is related to the structural characteristics of adipocytes, including common absence of adipocyte nuclei in the plane of sections and typical composition of large unstained cytoplasm and thin cell membrane seemingly joining with that of adjacent adipocytes.

We compared the usability and measurement results of our QuPath based system without using any manual correction to those with Adipocyte Tools plugin in ImageJ with manual correction of any misdetections. The latter effectively produces detections equivalent to full manual measurement and serves as a reliable reference for size measurements. With QuPath, it is possible to delete failed detections and correct them by manually annotating adipocytes. The types of misdetections observed included conjoined cells, misdetection of cell walls due to spill-over staining, or sometimes, misinterpretation of the space between the cells as an adipocyte. The notion that the QuPath based method without any manual correction produced a slightly smaller area and hence, smaller diameter for measured adipocytes may in part relate to this. However, measuring larger areas with thousands of cells and multiple samples and fixing all misdetections one by one would easily be time-consuming and may not be feasible in terms of the labour required. Even so, our comparison of adipocyte detection between Adipocyte Tools and QuPath indicates that the quality of samples plays an essential role in both methods and probably for any other software as well if manual correction is not applied. Fragmentation of the cells during sample preparation may result in faulty detections, as other authors have also observed [21]. Miscut or overly thick sections may also cause excessive staining leading to aberrations in adipocyte detection.

The QuPath based method provided only a slightly smaller cell area and diameter measured as compared with measurements performed with Adipocyte Tools with manual correction. The inter-method analysis showed good to excellent consistency and correlation between the two methods. In practical applications, such as research focusing on alterations of tissue morphology due to intervention or treatment of obesity or adipose dysfunction, such negligible differences in absolute values are unlikely to cause any bias in the analyses. Furthermore, if necessary, measurements obtained by QuPath can be corrected to correspond to those by Adipocyte Tools with manual correction with just a simple coefficient. As a substitute, the QuPath based method offers a feasible means to pursue measuring significantly larger adipocyte areas, thus potentially evading sampling biases such as choosing sample site or decreased accuracy associated with small sample areas [16]. The results presented here show that average adipocyte size diminishes and size deviation increases slightly when measuring larger areas of the sample or even whole-slide samples, indicating that cells’ size may vary depending on their location, or cells may even form clusters of even size, thus further emphasizing the role of site choosing when using smaller sample areas.

Lastly, we applied all measurement methods utilized here to samples from obese subjects with BMI > 35 kg/m^2^ to test the protocol in an experimental setting and to compare the results to those presented in the literature. Mean FCS diameter seems to correlate with BMI and reach a plateau around 75–80 µm at BMI 25 kg/m^2^ and 35 kg/m^2^ for men and women, respectively [6]. Thus, in this set of subjects, the BMI-dependent rise of FCS is expected to have reached a plateau. Our measurements with different methods including the QuPath based method introduced here correspond with the values presented in the literature [6].

Recently, 16 and Tratwal et al.^21^ reported QuPath plugins which seem to operate with good consistency as tested on mice adipose tissue. However, in the protocol of Maguire et al., the actual analysis was performed with ImageJ, not with QuPath. Tratwal et al. used an earlier version of QuPath (version 0.1.4) where the Pixel classifier option was not yet available, and recognition of adipocytes was described to base on a script using the ImageJ/Fiji-extension. However, our QuPath based method requires no installation of additional plugins, knowledge of programming or script writing, and may thus be more accessible to entry-level users.

In conclusion, we describe here a method and consistency using the free open-source software QuPath and its inbuilt tools for quantifying adipocyte count and parameters in human H&E stained tissue samples. The method poses no need for external plugins or knowledge of scripts and is applicable for substantially larger areas than traditional micrograph-based analyses, and with some more effort, even for whole slides consisting of at least up to 12,000 adipocytes, and probably even more.

## Data Availability

This report describes a novel method of adipocyte size and count measurement. The series of histological samples here were only used to demonstrate the functionality and the consistency of this novel method as compared with an established alternative method (ImageJ + Adipocyte Tools with or without manual correction). Accordingly, original images as such are irrelevant in respect to main findings as the method can be applied and its performance compared by using any H&E stained adipose tissue samples. Furthermore, the scatterplots included provide detailed data of all measurements performed and there is no additional raw data to be shared.
